# Erratum: Thermal Expansion of Platinum and Platinum-Rhodium Alloys

**DOI:** 10.6028/jres.091.037.errata2014

**Published:** 2014-11-10

**Authors:** RE Edsinger, ML Reilly, JF Schooley

**Affiliations:** National Institute of Standards and Technology, Gaithersburg, MD, 20899

J. Res. Natl. Inst. Stand. Technol., Volume 91, Number 6, November-December 1986, p. 333 http://dx.doi.org/10.6028/jres.091.037

**Erratum:** Equation (22) contains an error. It should read

$$100\;\varepsilon (t,\;0{}^{\circ}C)=8.79\times 10^{-4}t+0.805\times 10^{-7}\;t^{2}.$$ (22)

Also, the data presented in Table 12 under the column Day and Sosman Eq. (22) should be evaluated by using the corrected Eq. (22) above.

